# The *H19-PEG10/IGF2BP3* axis promotes gastric cancer progression in patients with high lymph node ratios

**DOI:** 10.18632/oncotarget.20209

**Published:** 2017-08-05

**Authors:** Satoru Ishii, Keishi Yamashita, Hiroki Harada, Hideki Ushiku, Toshimichi Tanaka, Nobuyuki Nishizawa, Keigo Yokoi, Marie Washio, Akira Ema, Hiroaki Mieno, Hiromitsu Moriya, Kei Hosoda, Mina Waraya, Hiroshi Katoh, Masahiko Watanabe

**Affiliations:** ^1^ Department of Surgery, Kitasato University School of Medicine, Sagamihara, Japan; ^2^ Department of Surgery, Kitasato University Medical Center, Saitama, Japan; ^3^ Department of Surgery, Sagamino Hospital, Sagamihara, Japan

**Keywords:** gastric cancer, lymph node ratio, H19, PEG10, IGF2BP3

## Abstract

We previously demonstrated that the lymph node ratio (LNR) is a prognostic factor associated with *EGFR* expression, among first priority genes amplified or overexpressed in cancer. Here, we investigated the associations between high LNR and second, third, and fourth priority genes. We performed mRNA expression microarray analysis of tumor tissue from patients with stage III gastric cancer and high or low LNRs. Candidate high LNR-associated genes were further evaluated in 39 patients with stage III gastric cancer. The functional relevance of these genes was evaluated in gastric cancer cell lines. We focused on five genes: *H19*,*PEG10*, *IGF2BP3*, *CD177,* and *PGA3*. *H19* and *PEG10* were confirmed as high LNR-associated genes. *H19*, *PEG10*, and *IGF2BP3* were found to promote each other’s expression. Knocking down *H19* or *PEG10* using RNAi decreased cell proliferation, invasion, anchorage-independent growth, and chemoresistance. These genes had a mutual relationship in MKN7 cells. *H19* knockdown decreased expression of epithelial-mesenchymal transition-associated genes in MKN74 cells to suppress transformation. Thus, *H19* promotes epithelial-mesenchymal transition in gastric cancer and is a potential therapeutic target.

## INTRODUCTION

Gastric cancer (GC) is the third leading cause of cancer-related death in men and the fourth in women worldwide. There were approximately 951,600 new GC cases and 723,100 GC-related deaths in 2012 [1]. TNM stage is the most important prognostic factor in GC [2–4]. However, there is variability in clinical course among patients with the same stage disease [5]. Biomarkers are important to predict which patients have a high risk of recurrence.

We recently demonstrated that lymph node ratio (LNR) is an independent prognostic factor in pathological stage III (pStage III) GC patients. Patients with 14^th^ JGCA / 7^th^ UICC pStage IIIC GC and a high LNR had the worst prognosis [6, 7]. The LNR is the ratio of the number of metastatic lymph nodes to the number of dissected lymph nodes [8]. Multi-variate analysis indicated LRN has prognostic value in advanced GC, regardless of stage, and is a more accurate prognostic marker than N stage [5, 9–12]. LNR could also adjust stage migration [5]. The LNR can indicate the success of lymph node dissection and reflect patient immune response [6].

A high LNR is correlated with a poor prognosis in advanced GC patients. We previously found that a high LNR was associated with *EGFR* expression in advanced GC [13]. However, our study only focused on genes in the first priority group. Here, we investigated the expression of genes in the second, third, and fourth priority groups. Our results indicate the long non-coding RNAs (lncRNAs) *H19* and *PEG10* are associated with a high LNR. We also demonstrated a mutual relationship between *H19* and *PEG10* expression, and confirmed that these genes were associated with GC aggressive behavior *in vitro*.

## RESULTS

### Verification of the prognostic significance of the LNR in pStage IIIC GC patients

**Figure 1:**
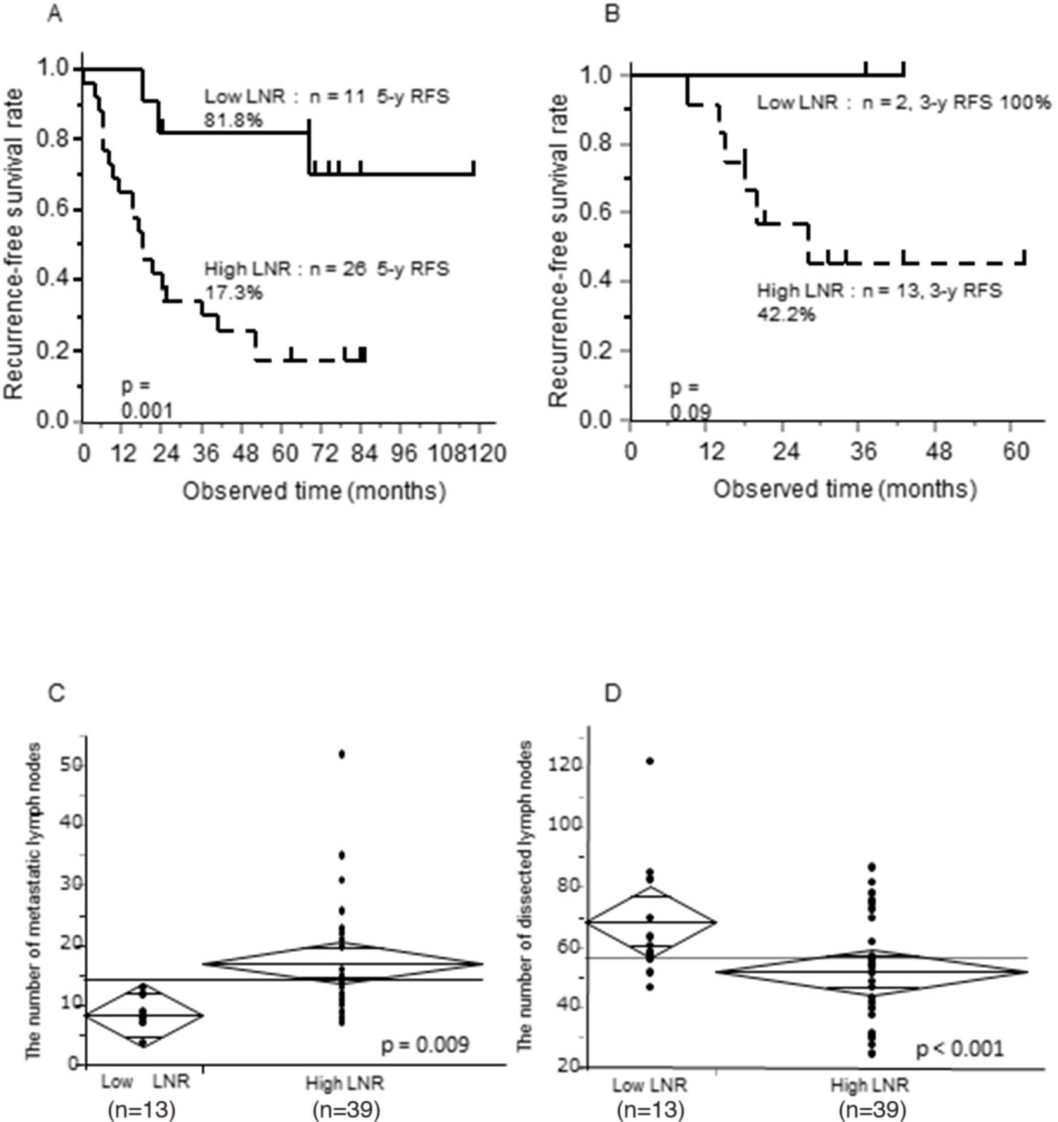
Prognostic significance of the LNR, 5-year RFS according to LNR status, and relationship between the LNR and number of metastatic or dissected lymph nodes in pStage IIIC GC patients (**A**, **B**) Kaplan Meier curves of 5-year RFS according to low or high LNR (LNR cut-off, 16.7%) (A) in pStage IIIC GC patients treated at our institution between 2000 and 2010, and (B) in pStage IIIC GC treated between 2011 and 2015. (**C**, **D**) T-tests between the LNR and (C) the number of metastatic lymph nodes or (D) the number of dissected lymph nodes.

We previously reported that the LNR was a strong independent prognostic factor in patients with stage II/III advanced GC who underwent curative gastrectomy and received postoperative S-1 adjuvant therapy at our institution between 2000 and 2010 [6, 7]. However, this prognostic relevance was restricted to pStage IIIC GC patients. Here, it was evaluated in 37 patients with pStage IIIC GC who were treated at our institution between 2000 and 2010 (Figure 1A, p = 0.001, Supplementary Table 1, 5-year recurrence-free survival [RFS]; LNR < 16.7%, 81.8%; LNR ≥ 16.7%, 17.3%). The results were validated in an independent cohort of 15 similar patients who were treated between 2011 and 2015 (Figure 1B, p = 0.09, Supplementary Table 2). The 5-year RFS rate was 100% in the low LNR group (LNR < 16.7%) and 42.2% in the high LNR group (LNR ≥ 16.7%).

We assessed the relationship of the LNR with the number of either dissected or metastatic lymph nodes in pStage IIIC GC patients (Figure 1C and 1D). There was a positive correlation between the number of metastatic lymph nodes and the LNR (p = 0.009), while there was a negative correlation between the number of dissected lymph nodes and the LNR (p < 0.001). These results suggested that the LNR reflected tumor aggressiveness, the host immune response, or stage migration in pStage IIIC GC patients.

### Selection of candidate high LNR-associated genes associated in pStage III GC patients

**Figure 2:**
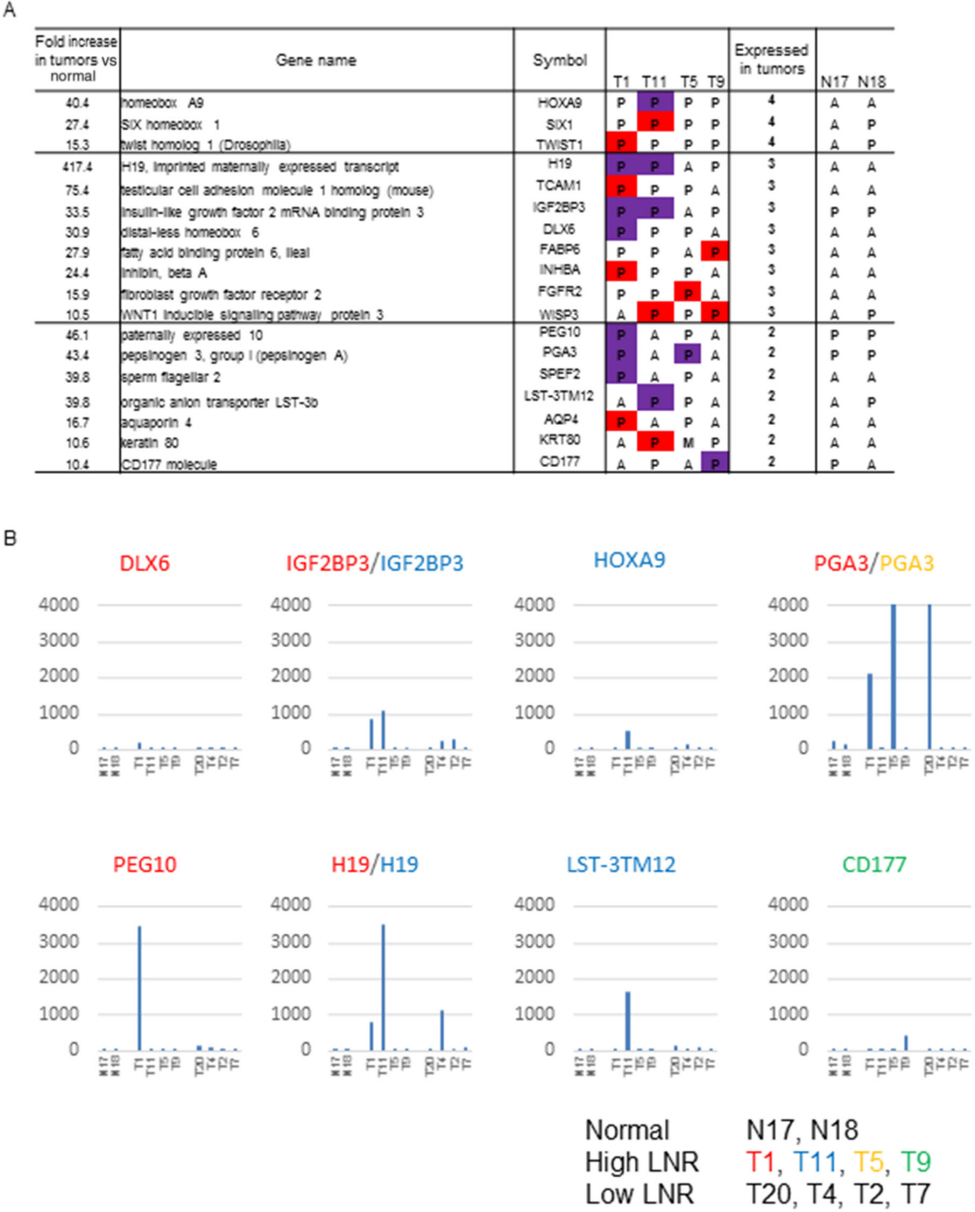
Genes identified as strongly associated with a high LNR in the expression microarray **(A)** Genes in the second, third, and fourth priority groups showing a high expression in tumors. The table shows tissue specimens with raw expression of 100 to 200 in red, and ≥ 200 in purple, in the comparison of GC patients with high and low LNRs. P: present, M: marginal, A: absent in expression by microarray. **(B)** Gene expression values of candidate high LNR-associated genes with FI > 200 in tumor tissue from GC patients with high LNRs (n = 4) (T1, T11, T5, T9; middle group of patients), low LNRs (n = 4) (T20, T4, T2, T7; patient group on the right), and controls (non-cancerous tissue; n = 2) (N17, N18; first two patients). Raw microarray data (uncorrected). The β-catenin signal was reproduciblefrom approximately 4,000 to 10,000. The color of the gene name corresponds to each patient with a high LNR.

Among the second, third, and fourth priority genes identified in our previous study as strongly associated with high LNR, 18 genes with high expression (raw expression values > 100) were not further evaluated (Figure 2A). Genes in the second, third, and fourth priority groups had raw expression values > 100 in at least one patient in the Affymetrix microarray data. Signals > 100 were reproducibly detected by RT-PCR (Figure 2A) [14]. Data in the red columns represents raw expression values between 100 and 200. Data in the purple columns represents values > 200. We presented an mRNA microarray data to represent the expression of candidate genes with raw expression values > 200 (Figure 2B).

The final five candidate genes (*PEG10*, *H19*, *IGF2BP3*, *PGA3,* and *CD177*) were selected for the following reasons: *PEG10* showed the highest expression in a T1 (high LNR) patient, *IGF2BP3* and *H19* showed high expression in both T1 and T11 (high LNR) patients, *PGA3* showed high expression in both T1 and T3 (high LNR) patients, and *CD177* showed the highest expression in a T9 (high LNR) patient. *H19* and *PGA3* showed high expression in T4 and T20 (low LNR) patients, respectively. However, they were selected because of their high frequency of expression in patients with high LNRs (2/4, 50% vs. 1/4, 25%). These five genes were considered candidate high LNR-associated genes.

### Validation of candidate high LNR-associated genes in advanced GC patient tissue samples

**Figure 3:**
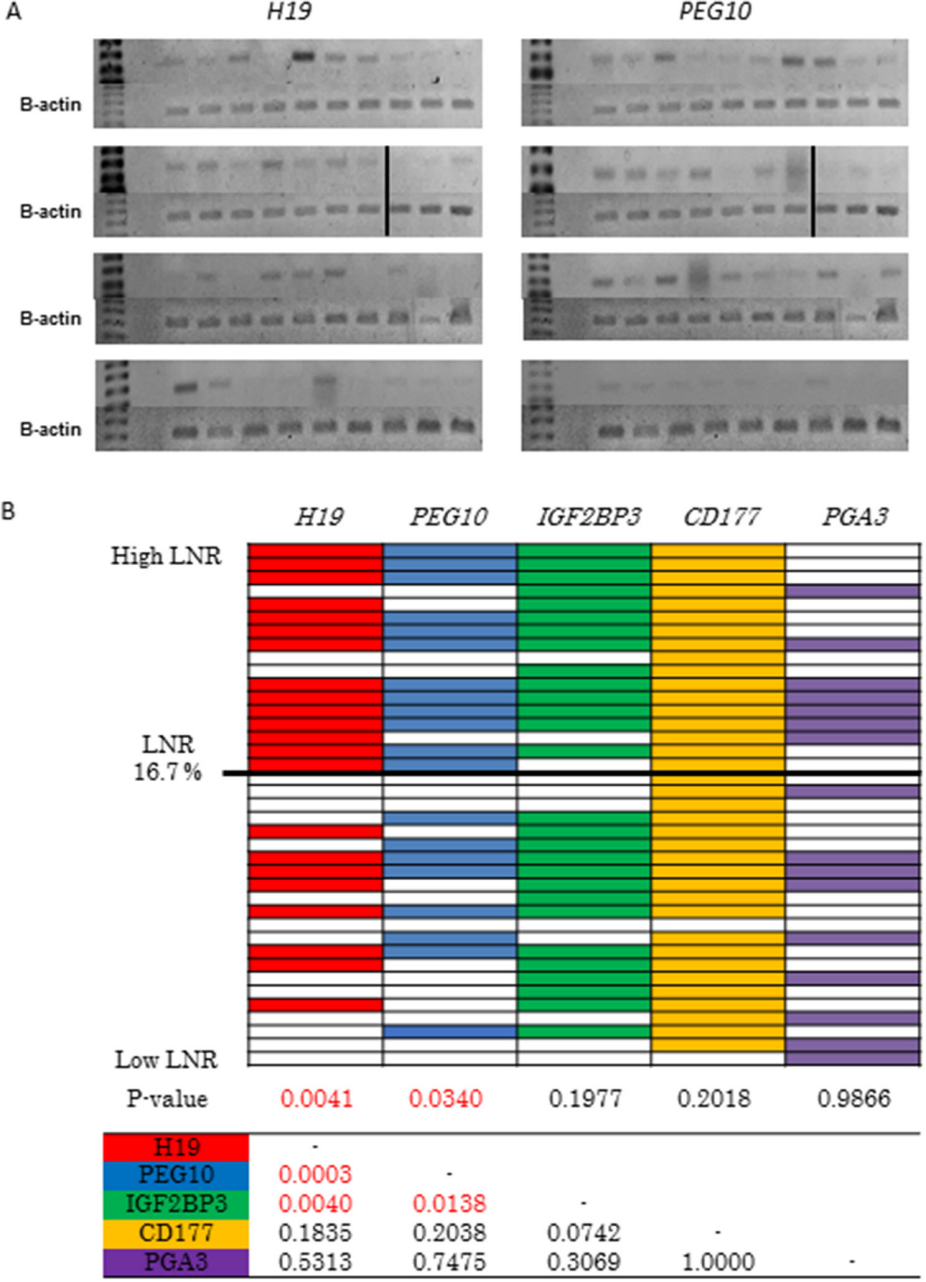
Selection of candidate high LNR-associated genes **(A)** Analysis of *H19* and *PEG10* expression in pStage III GC tissue samples using RT-PCR. Patients are shown in descending order from the upper left to the lower right according to LNR. **(B)** RT-PCR analysis of the expression of the five candidate high LNR-associated genes in pStage III GC. Patients are shown in descending order from top to bottom according to LNR. Color indicates positive and white indicates negative. P values were calculated using Fisher's exact or chi-square tests. The table shows the relationship between the five candidate genes.

We next investigated the relationship between the expression of *PEG10*, *H19*, *IGF2BP3*, *PGA3*, and *CD177* and LNR in 39 patients with pStage III GC who underwent curative gastrectomy at our institution between 2008 and 2011 using RT-PCR. Of the 39 patients, 17 (43.6%) had a high and 22 (56.4%) had a low LNR (Supplementary Table 3). RT-PCR analysis revealed higher *H19* and *PEG10* expression in the high compared to low LNR group (p = 0.0041 and p = 0.034, respectively) (Figure 3A and 3B). Similar expression of *IGF2BP3*, *PGA3*, and *CD177* was observed in the high and low LNR groups (Figure 3B). We observed frequent co-expression of *H19* and *PEG10* (16/39, 41%) (Figure 3B). Mutual relationships were observed between *H19* and *PEG10*, *H19* and *IGF2BP3*, and *PEG10* and *IGF2BP3* expression (Figure 3B). These correlations in gene expression could be due to selection of genes with high expression in the high LNR group. Thus, *H19* and *PEG10* were identified as high LNR-associated genes, and their expression was positively correlated in pStage III GC.

### Epigenetic regulation of the *H19*-*PEG10* axis

**Figure 4:**
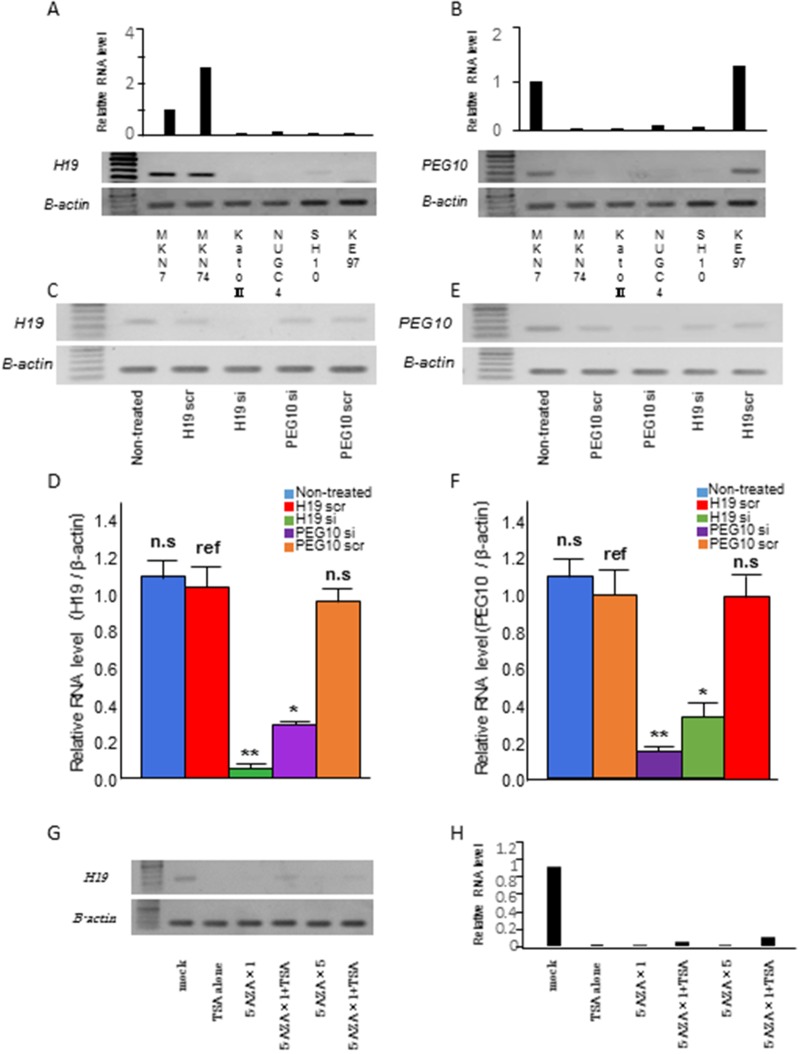
Relationship between *H19* and *PEG10* in GC cells **(A, B)** Analysis of (A) *H19* and (B) *PEG10* expression in six GC cell lines using qRT-PCR (top) and RT-PCR (bottom). (**C**, **D**, **E**, and **F**) MKN7 cells that express both *H19* and *PEG10* were transfected with (C) *H19* siRNA (*H19* si), (D) *H19* scrRNA (H19 scr), (E) *PEG10* siRNA (*PEG10* si), or (F) *PEG10* scrRNA (*PEG10* scr). *H19* and *PEG10* RNA were analyzed using RT-PCR (top) and qRT-PCR (bottom) 48 h post-transfection. T tests were performed to compare the mean values of each RNA between scrambled RNA and siRNA. The numbers represent the mean ± standard deviation (s.d.; n = 3). *p < 0.05, ** p < 0.01. ref: reference, n.s.: not significant. (**G** and **H**) Epigenetic regulation of *H19* expression in MKN7 cells. *H19* expression was analyzed using RT-PCR (G) and qRT-PCR (H) in MKN7 cells treated with the 5-Aza-dC and/or TSA. *H19* expression was analyzed after treatment with 300 nM TSA alone, 1 μM 5-Aza-dC, 1 μM 5-Aza-dC and 300 nM TSA, 5 μM 5-Aza-dC, 5 μM 5-Aza-dC and 300 nM TSA were added in order from left to right.

*H19* and *PEG10* mRNA expression was investigated in six GC cell lines using RT-PCR and qRT-PCR. *H19* was expressed in MKN7 and MKN74 cells, while *PEG10* was expressed in MKN7 and KE97 cells (Figure 4A and 4B).

We knocked down endogenous *H19* and *PEG10* expression in MKN7 cells using siRNA. Knockdown was confirmed using both RT-PCR (Figure 4C and 4E) and qRT-PCR (Figure 4D and 4F). Interestingly, *H19* knockdown reduced *PEG10* mRNA expression. Conversely, *PEG10* knockdown reduced *H19* mRNA expression. These data suggested that *H19* and *PEG10* promoted each other’s expression. *PEG10* was not expressed in MKN74 cells despite high expression of *H19*, while *PEG10* was highly expressed in KE97 cells, which did not express *H19*. These data indicated that the correlation between *H19* and *PEG10* may not be present in all cancer cell lines.

*H19* expression is controlled by methylation of a differentially methylated region (DMR). Therefore, we investigated whether *H19* expression was regulated by demethylation and/or histone deacetylation. *H19* expression in MKN7 cells was reduced by treatment with a demethylating agent (5-Aza-dC) or histone deacetylase (trichostatin A [TSA]) (Figure 4G and 4H) through an indirect mechanism.

### *H19* knockdown suppresses proliferation, invasion, and anchorage-independent growth, and decreases chemo-resistance in GC cell lines

**Figure 5:**
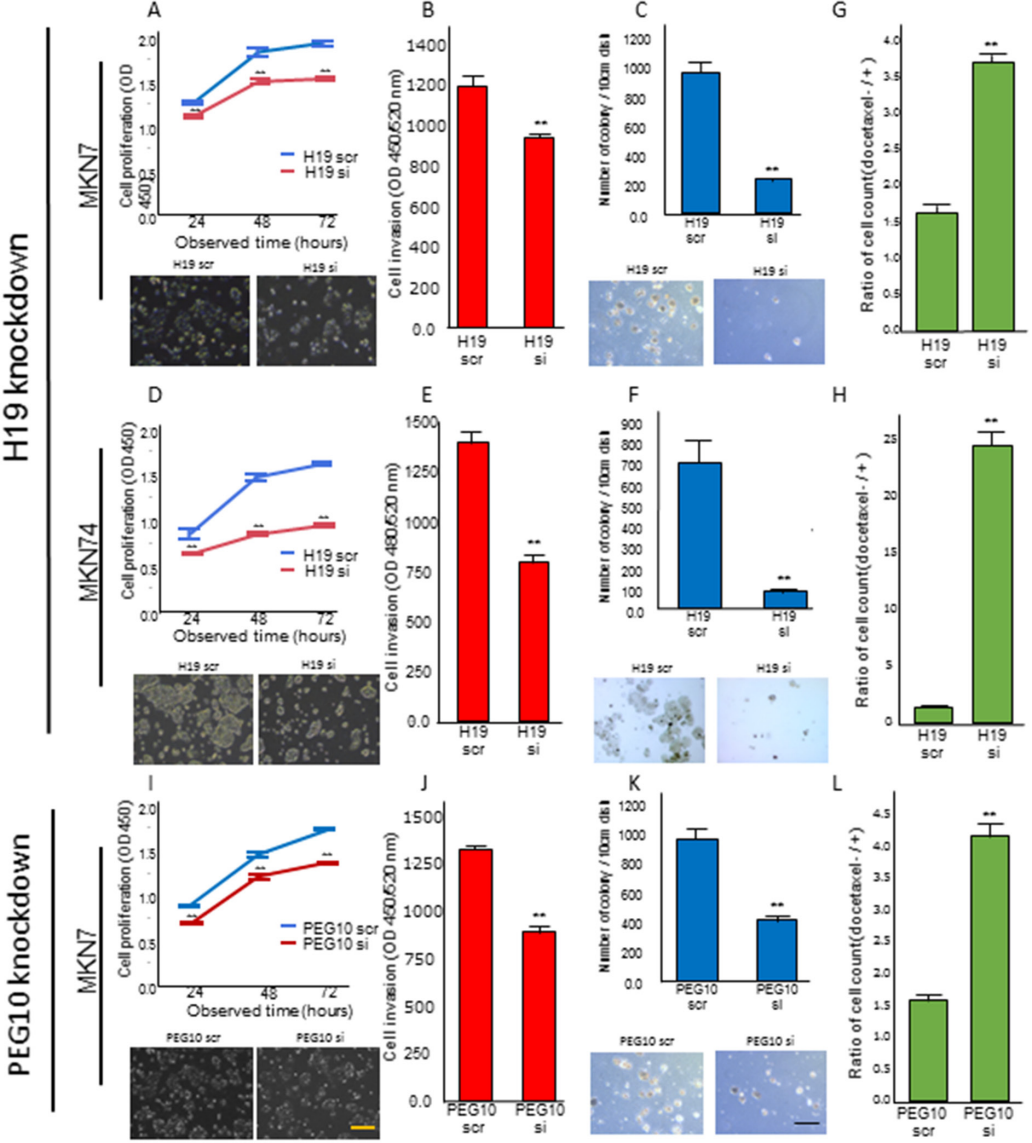
*H19* or *PEG10* knockdown decreased proliferation, invasion, anchorage-independent growth, and chemo-resistance **(A, B,** and **C)** MKN7 cells were transfected with the indicated *H19* siRNA, and analyzed using (A) proliferation, (B) invasion, and (C) anchorage-independent growth assays. The numbers represent the mean ± s.d. (n = 3). **p < 0.01. **(D, E,** and **F)** MKN74 cells were transfected with the indicated *H19* siRNA and analyzed using (D) proliferation, (E) invasion, and (F) anchorage-independent growth assays. The numbers represent the mean ± s.d. (n = 3). **p < 0.01. **(I, J,** and **K)** MKN7 cells were transfected with the indicated *PEG10* siRNA, followed by (I) proliferation, (J) invasion, and (K) anchorage-independent growth analyses. The numbers represent the mean ± s.d. (n = 3). **p < 0.01. Representative phase-contrast microscopy images for two independent experiments are shown in A, C, D, F, I, and K. The length of the black and yellow lines are 100 µm and 25 µm, respectively. (**G**, **H**, and **L**) Measurement of the number of viable cells 48 h after 0.25 mM docetaxel treatment in (G) *H19* or (L) *PEG10* knockdown MKN7 cells, and (H) *H19* knockdown MKN74 cells. The numbers represent the mean ± s.d. (n = 3). **p < 0.01.

We investigated the effects of *H19* or *PEG10* knockdown in MKN7 and MKN74 cells, which have high *H19* mRNA expression, on transformation phenotypes. *H19* knockdown in MKN7 cells decreased proliferation, invasion, and anchorage-independent growth compared to control cells transfected with scramble in WST-1, matrigel invasion, and colony formation assays, respectively (Figure 5A, 5B, and 5C, respectively). Similarly, *H19* knockdown in MKN74 cells decreased proliferation, invasion, and anchorage-independent growth versus controls (Figure 5D, 5E, and 5F, respectively).

We next investigated the effect of *H19* knockdown on the chemo-sensitivity of MKN7 and MKN74 cells using the microtubule inhibitor docetaxel. Docetaxel is frequently used as a chemotherapeutic for patients with advanced GC [15–17]. In both MKN7 and MKN74 cells, *H19* knockdown resulted in reduced cell viability and enhanced chemo-sensitivity (Figure 5G and 5H).

### *PEG10* knockdown suppresses proliferation, invasion, and anchorage-independent growth, and decreases chemo-resistance in GC cells

We knocked down *PEG10* in MKN7 cells, which highly express both *PEG10* and *H19.* These genes promoted each other’s expression in MKN7 cells. *PEG10* knockdown in MKN7 cells decreased proliferation, invasion, and anchorage-independent growth in WST-1, matrigel invasion, and soft agar assays *in vitro*, respectively (Figure 5I, 5J, and 5K). Furthermore, *PEG10* knockdown enhanced the sensitivity of MKN7 cells to docetaxel, similar to the effects of *H19* knockdown, leading to reduced cell viability (Figure 5L).

### An *H19*-*PEG10* / *IGF2BP3* axis promotes the malignant phenotype of GC cells

**Figure 6:**
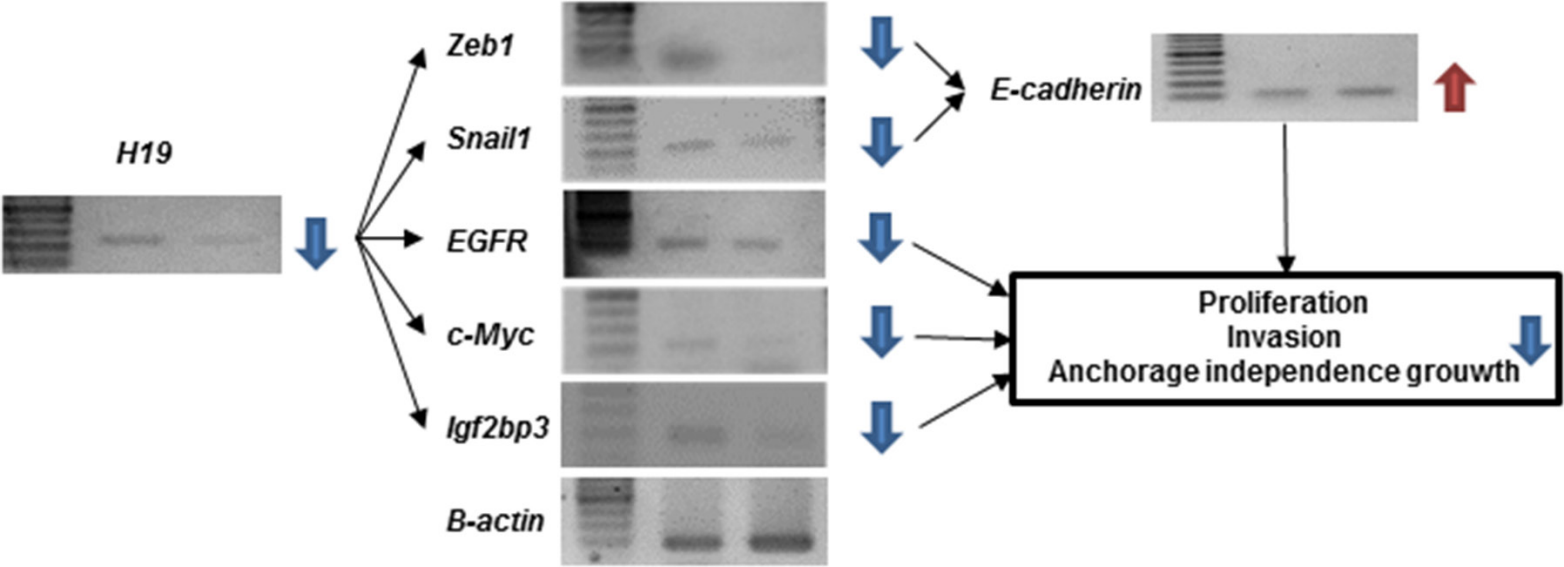
Analysis of downstream signaling and malignant phenotypes following *H19* knockdown in MKN74 cells Downstream signaling pathway components involved in malignant behavior were analyzed in MKN74 cells after *H19* knockdown by RT-PCR. Reduced *H19* expression led to a decrease in *Zeb1*, *Snail1*, *EGFR*, *c-Myc,* and *IGF2BP3* expression. An increase in *E-cadherin* expression.

We hypothesized that the relationship between *H19* and *PEG10* in MKN7 cells could impact transformation in GC cells. Although *H19* did not induce *PEG10* expression in MKN74 cells, *H19* knockdown suppressed transformation (Figure 5D, 5E, 5F, and 5H). We therefore investigated the molecular mechanism by which *H19* knockdown suppressed transformation in MKN74 cells. *H19* acts as a sponge for *let-7, miR-200,* and *miR-34,* which results in the induction of *IGF2BP3*, *c-Myc*, and *Zeb1* expression, respectively. This leads to induction ofepithelial-mesenchymal transition (EMT) as a consequence of the suppression of *E-cadherin* expression via increased expression of *Zeb1* and *Snail1* [18–27]. We examined whether *H19* knockdown altered the expression of *IGF2BP3*, *c-Myc*, *Zeb1*, *Snail1*, or *E-cadherin* in MKN74 cells (Figure 6). *H19* knockdown in MKN74 cells decreased the expression of *IGF2BP3*, *c-Myc*, *Zeb1*, and *Snail1,* and increased the expression of *E-cadherin* (Figure 6). *IGF2BP3* expression was correlated with *H19* expression (Figure 3B), and was generally associated with a high LNR in primary GC.

## DISCUSSION

The LNR can reflect both the number of metastatic and dissected lymph nodes [6–8]. It can adjust stage migration and surgical lymph node dissection. Our data indicate it predicts the prognosis of pStage IIIC GC patients (Figure 1) [6]. Stage migration is frequently observed in pStage IIIC GC patients. Thus, a high LNR is indicative of aggressive GC. *H19* expression was tightly correlated with high LNR. Additionally, high expression of *PEG10*, *IGF2BP3*, and *EGFR* [13] was correlated with high LNR. *H19* expression was associated with *PEG10* and *IGF2BP3* expression.

We demonstrated that *H19* and *PEG10* promote each other’s expression in GC cells. We investigated the role of the *H19*-*PEG10* axis in GC cell proliferation, anchorage-independent growth, invasion, and chemo-sensitivity. *H19* is involved in EMT and promotes cancer cell proliferation, invasion, and metastasis [28–38]. *H19* has two major functions: it acts as a reservoir of *miR-675* [33, 34], and it acts as a molecular sponge to regulate miRNA and protein availability [18].

Knockdown of *H19* resulted a large change in the expression of EMT-associated proteins. The *H19*-encoded *miR-675* promotes skeletal muscle differentiation and regeneration [34]. *MiR-675* promotes tumor progression by suppressing the expression of target genes including *Rb* [32], *RUNX1* [33], *TGFBI* [35], and *Twist1* [39]. *MiR-675* also promotes stabilization and activation of *EGFR* and *c-Met*, which enhances cell proliferation and migration [40]. The indirect association between *miR-675* and *EGFR* supports our finding that *EGFR* expression decreased after *H19* knockdown. Thus, targeting *H19*, in addition to EGFR, may improve the prognosis of patients with advanced GC and a high LNR. Future studies will examine *EGFR* and *H19* expression in GC patients with high LNRs.

*H19* antagonizes a variety of miRNAs that are involved in metastasis [18]. For example, it antagonizes *let-7*, a potent tumor suppressor [20, 21], which post-transcriptionally represses the expression of metastasis-promoting genes including *c-Myc* and *IGF2BP3* [22]. *H19* also acts as a sponge for the *miR-200* family through a similar mechanism. The *miR-200* family suppresses *Zeb1/2 expression* (*Zeb1/2*-*miR-200* loop), which suppresses *E-cadherin* expression [23, 24]. In addition, *H19* suppresses *miR-34*, which suppresses *Snail1* expression (*Snail1*-*miR-34* loop) [25–27]. *H19* suppresses both negative feedback loops, thereby inhibiting mesenchymal-epithelial transition. Thus, *H19* plays an important role in EMT.

We also found that *H19* is an upstream regulator of *IGF2BP3* expression. Although *IGF2BP3* expression was not associated with LNR in the microarray analysis of pStage III GC patients, the frequency of expression was high (71.8%, 28/39), indicating a relationship with *H19* expression. *IGF2BPs* (1, 2, and 3) play important roles in RNA trafficking, stabilization, localization, and cell migration, particularly during the early stages of human and mouse embryogenesis [41, 42]. *IGF2BPs* are expressed in developing epithelia, the placenta, and muscle, but expression is undetectable in normal adult tissues [43]. *IGF2BP3* is re-expressed in several malignant tissues [44–49]. Indeed, *IGF2BP3* was shown to promote the association between the RNA-induced silencing complex with specific transcripts, and influence tumor-associated RNA regulation by modulating miRNA-mRNA interactions [50]. *IGF2BP3* is regulated by *let-7*, which in turn is sequestered by *H19* [22]. Thus, the *H19*-*IGF2BP3* axis may explain these molecular interactions. *IGF2BP3* is upregulated by *EGFR* [51], which promotes tumor cell migration and invasion by inducing expression of pre-migration/invasion genes [52–58]. *EGFR* also promotes aggressive phenotypes in tumor cells [59–62]. Although we only observed a relationship between the *EGFR* and *H19*-*IGF2BP3* and high LNR, we described the existence of a *let-7*-*IGFF2BP3* feedback loop mediated by the molecular sponge activity of *H19*, and regulation of *IGF2BP3* by *EGFR* and *miR-675*. These mechanisms may in part explain the aggressive behavior of GC cells.

We have identified an *H19*-*PEG10* axis in GC. *PEG10* has recently attracted attention regarding its potential role in cancer proliferation, invasion, and metastasis [63–68]. The effects of *H19* knockdown were similar to those of *PEG10* knockdown. *PEG10* is a paternally expressed gene located on human chromosome 7q21 [69]. Aberrant *PEG10* expression has been associated with various human malignancies [61–66]. *PEG10* stimulates cell proliferation through interaction with ALK1, which inhibits TGF-β signaling [70], or with *SIAH1*, an inducer of apoptosis [71, 72]. *PEG10* promotes cell cycle progression from G0/G1 and regulates *Snail* expression via TGF-β signaling [65]. *PEG10* also participates in a positive feedback loop with *c-Myc* [73]. *H19* was also reported to participate in a positive feedback loop involving *c-Myc* [19]. The involvement of *c-Myc* in the positive feedback loop including *H19* and *PEG10* may be part of the mechanism underlying *H19*-*PEG10* axis function. Knockdown of *H19* or *PEG10* induced the expression of EMT-related genes. Thus, the *H19*-*PEG10* axis should be considered a potential therapeutic target in GC.

*H19* overexpression has been reported in various human cancers including GC [74–78]. We observed differences in expression in GC cell lines (Figure 4A). *H19* is located on human chromosome 11p15.5. Expression is controlled by parental imprinting at the *IGF2*/*H19* locus (*H19* is maternally expressed) [79–81]. Imprinting of the *H19* gene is accomplished by methylation of the DMR in normal cells [79, 80]. CpG methylation of promoters is a dynamic process. We analyzed *H19* expression in MKN7 cells treated with 5-Aza-dC and TSA. *H19* expression was suppressed in MKN7 cells as a result of demethylation and/or histone deacetylation (Figure 4G). *H19* expression may be suppressed by reactivation of a tumor suppressor gene that regulates *H19*, and targeting this epigenetic regulation may be effective for the treatment of GC patients with *H19* overexpression.

*H19* was detected in blood samples from GC patients [82]. The abundance of *H19* in plasma suggests that it plays a systemic role in cancer progression. *H19* generates *miR-675*, which is an oncogenic miRNA [29–40]. Further studies are required to analyze the systemic distribution of *miR-675* in plasma as well as the distribution in the peritoneal cavity, which is the major site of GC recurrence. We demonstrated overexpression of *H19* in GC patients with aggressive disease and lymph node metastasis. These patients have a poor prognosis, even after standard postoperative adjuvant chemotherapy [6]. *H19* knockdown suppressed chemo-resistance, suggesting that *H19* expression is important for GC progression. *H19* expression in the tumor microenvironment is of systemic and local significance.

In summary, the *H19*-*PEG10*/*IGF2BP3* axis may explain the aggressive features of primary GC (i.e. invasion, metastasis, and chemo-resistance). Therapeutics that target this axis (and lncRNAs) could augment the effects of chemotherapy.

## MATERIALS AND METHODS

### Patient samples for validation of LNR as a prognostic factor

We first confirmed the relationship between LNR and prognosis in GC. We previously demonstrated that LNR could predict prognosis in pStage II/III GC patients who underwent curative gastrectomy and received S-1 adjuvant therapy. Prognostic stratification based on LNR was only possible in pStage IIIC GC patients [6]. We selected 37 patients with pStage IIIC GC who were treated at our institution between 2000 and 2010 [6]. We updated their prognosis and analyzed the prognostic value of the LNR (Supplementary Table 1). Additionally, 15 patients with pStage IIIC GC were enrolled between 2011 and 2015, and prognostic analysis performed (Supplementary Table 2). The LNR cut-off value was 16.7% [6].

### Tissue samples for selection and validation of candidate high LNR-associated genes

We analyzed tumor specimens from 39 patients with pStage III GC who underwent curative gastrectomy and received S-1 adjuvant chemotherapy at our institution between 2008 and 2011 (Supplementary Table 3). None of the patients received chemotherapy or radiotherapy prior to surgery. All tissue samples were collected at the Kitasato University Hospital and analyzed according to the Declaration of Helsinki. Written informed consent was obtained from all patients and healthy donors before sample collection. The study was approved by the Ethics Committee of Kitasato University.

### Microarray gene expression analysis

We previously described a method of mRNA expression microarray analysis of candidate high LNR-associated gens [13]. Here, we comprehensively analyzed genes in the second, third, and fourth priority groups, which included genes that had high expression in two, three, or four tumors, respectively.

### Cell lines and culture

We analyzed six GC cell lines (MKN7, MKN74, Kato-III, NUGC4, SH-10-TC, and KE97). The MKN7 cell line was obtained from the Cell Resource Center for Biomedical Research Institute of Development, Aging, and Cancer at Tohoku University (Sendai, Japan). The other five GC cell lines were purchased from the RIKEN BioResource Center (Ibaraki, Japan). These cell lines represent the two main types of GC [83], the intestinal type (MKN7 and MKN74 cells) and the diffuse type (Kato-III, SH-10-TC, KE97, and NUGC4 cells) [84–86]. All media contained 10% fetal bovine serum and penicillin-streptomycin (GIBCO, Carlsbad, CA, USA).

### RNA extraction, reverse transcription PCR (RT-PCR), and quantitative RT-PCR (qRT-PCR)

RNA extraction from gastric tissue and cell lines, and reverse transcription to generate cDNA were performed as described previously [13]. RT-PCR was performed on equal amounts of cDNA. The qRT-PCR reactions were conducted in triplicate. Target gene levels were normalized to β-actin and analyzed using the C_T_ method. The PCR conditions and sequences of the primer and probes are shown in Supplementary Table 4.

### *H19* and *PEG10* siRNA transfection

The siRNAs targeting human *H19* and *PEG10* were purchased from Sigma-Aldrich (St Louis, MO, USA). The *H19* siRNA sequences were as follows: sense, 5’-ccaacaucaaagacaccau-3’; antisense, 5’-auggugucuuugauguugg-3’ [87]. The *PEG10* siRNA sequences were as follows: sense, 5’-gucgcugucugcucugauu dt-3’; antisense, 5’-aaucagagcagacagcgac-3’ [68]. We used MISSION siRNA Universal Negative Control (Sigma-Aldrich) as a control. Cells were seeded in 10 cm dishes overnight and grown to 30–40% confluence. Cells were then transfected with 600 pmol of siRNA using the Lipofectamine 2000 (InvitrogenLife Technologies, Carlsbad, CA, USA) in OPTI-MEM medium (GIBCO) according to the manufacturer’s instructions. After 48 h, the cells were harvested and analyzed by RT-PCR and qRT-PCR.

### Cell proliferation and invasion assays

MKN7 and MKN74 cells were seeded at a density of 3.0 × 10^3^ cells/well and 4.0×10^3^ cells/well, respectively, in 96-well plates for cell proliferation assays. The cells were transfected with the relevantoligonucleotides, and cell proliferation quantified after 24, 48, and 72 hours using the Premix WST-1 Cell Proliferation Assay System (Takara Bio, Tokyo, Japan) according to the manufacturer’s protocol. Invasion assays were performed by measuring the ability of transfected cells to migrate through a Transwell chamber using the CytoSelect™ 96-Well Cell Invasion Assay (Basement Membrane, Fluorometric Format) (Cell Biolabs, Inc., San Diego, CA, USA) according to the manufacturer’s protocol. The fluorescence intensity was measured by the absorbance at 450 nm in the proliferation assays and at 450 nm/520 nm in the invasion assays using a microplate reader (Molecular Devices, LLC. Sunnyvale, CA, USA).

### Anchorage-independent colony formation assays

Anchorage-independent cell growth was analyzed by plating 0.36% top agarose (Bacto™ Agar, Becton, Dickinson and Company, Franklin Lakes, NJ, USA) containing 1.0 × 10^5^ cells on a surface of 0.72% bottom agarose in 6-well plates [88]. Cells were fed weekly by overlaying fresh soft-agar solution, and colonies imaged after 2–3 weeks of incubation. Two independent experiments were performed and each experiment was performed in triplicate.

### Chemo-sensitivity assays

Chemo-sensitivity assays were performed with docetaxel (Taxotere, Sanofi), which is useful as a single-agent or combination chemotherapy for the treatment of advanced GC [89, 90]. Docetaxel was administered at a final concentration of 0.25 µM following siRNA transfection. After 48 h of docetaxel treatment, cells were detached using Trypsin-EDTA (GIBCO) and viable cells counted using a Countess automated cell counter (Invitrogen Life Technologies).

### Treatment of MKN7 cells with 5-Aza-dC and TSA

In order to investigate whether the expression of *H19* was regulated by methylation of a DMR, MKN7 cells were treated with 1 µM or 5 µM 5-Aza-dC, a demethylating agent, (Sigma-Aldrich) dissolved in 50% acetic acid (Wako pure Chemical Industries, Osaka, Japan) once every 24 h for 4 days. Controls were mock-treated with an equal volume of PBS (GIBCO) once every 24 h for 4 days. In addition, 300 nM of the histone deacetylase inhibitor TSA (Sigma-Aldrich) was added to the cells for the final 24 h. Cells were detached on day 5 using Trypsin-EDTA and mRNA extracted using the RNeasy Mini Kit (Qiagen) for gene expression analysis [91].

### Statistical analysis

Categorical variables were analyzed using χ^2^ or Fisher’s exact tests. Differences in continuous variables between groups were evaluated using analysis of variance (ANOVA). Cumulative, 5-year RFS was estimated using the Kaplan-Meier method, and statistical differences were analyzed using log rank tests. RFS was measured from the date of surgery to that of recurrence or the last follow-up. A p < 0.05 was considered statistically significant. Factors with p < 0.05 on univariate analysis were subjected to multivariate analysis using a Cox proportional hazards model to identify independent prognostic factors. All analyses were performed using JMP version 11.1 (SAS Institute Japan, Tokyo, Japan).

## SUPPLEMENTARY MATERIALS TABLES









## Abbreviations

LNR: lymph node ratio
GC: gastric cancer
pStage: pathological stage
lncRNA: long non-coding RNA
miR: micro-RNA
mRNA: messenger RNA
RT-PCR: reverse transcription polymerase chain reaction
qRT-PCR: quantitative reverse transcription polymerase chain reaction
siRNA: small interfering RNA
RFS: recurrence free survival
ANOVA: analysis of variance
5-Aza-dc: 5-aza-2’-deoxycytidine
TSA: trichostatin A
DMR: differentially methylated region
EMT: epithelial mesenchymal transition
