# Supplementary material for: The H19-PEG10/IGF2BP3 axis promotes gastric cancer progression in patients with high lymph node ratios
Source: Oncotarget. 2017 Aug 5;8(43):74567–81. doi: 10.18632/oncotarget.20209 (PMC5650363; doi:10.18632/oncotarget.20209)
Supplement: Supplementary file 1 [file oncotarget-08-74567-s001.pdf]

The *H19-PEG10/IGF2BP3* axis promotes gastric cancer progression in patients with high lymph node ratios

SUPPLEMENTARY MATERIALS

Supplementary Table 1: Clinicopathological characteristics and recurrence-free survival or lymph node ratio in pStage IIIC gastric cancer patients from 2000 to 2010

See Supplementary File 1

Supplementary Table 2: Clinicopathological characteristics and recurrence-free survival or lymph node ratio in pStage IIIC gastric cancer patients from 2011 to 2015

See Supplementary File 2

Supplementary Table 3: Clinicopathological characteristics and recurrence-free survival in 39 patients with stageIII gastric cancer

See Supplementary File 3

Supplementary Table 4: PCR sequences of primers and fluorescent probe used in this study

Methods	Gene	Forward primer sequence (5' to 3')	fluorescent probe (5' to 3')	Reverse primer sequence (5' to 3')
RT-PCR/qRT-PCR	H19	aaagacaccatcggaacagc	acatcatcccagagctgagc	agagtcgtggaggctttgaa
RT-PCR/qRT-PCR	PEG10	tgcttctggcaacttcattg	acccagctttcatgatggaa	tcaaatgacagcacctctcg
RT-PCR	IGF2BP3	cagttcaaggctcagggaag		ggtcacgagggacaacaact
RT-PCR	PGA3	ttaccgtcgagggttactgg		tccattgatggtgaagacga
RT-PCR	CD177	ggcctggagtctctcacttg		ccccagtgttgctgttaggt
RT-PCR	CDH1 (E-cadherin)	tcatgagtgtcccccggtat		tcttgaagcgattgccccat
RT-PCR	EGFR	cgctaccttgtcattca		tgcactcagagagctcagga
RT-PCR	Zeb1	aacagttggtttggtgt		cttcacccatacaacaaggt
RT-PCR	Snail1	gtttaccttccagcagccct		gagcctttcccactgtcctc
RT-PCR/qRT-PCR	β-actin	tcacccacactgtgcccatctacga	ggacttcgagcaagagatgg	cagcggaaccgctcattgccaatgg

*1 : RT-PCR was done at 95°C for 3 min followed by 27 cycles at 95°C for 1 min, 60°C for 1 min, 72°C for 1 min, and final extension at 72°C for 10 min 1µl dNTP mixture, 1.5µl MgCl2 , 0.2µmol/l each primer and0.2µl Platinum Taq DNA polymerase.
*2 : qRT-PCR was done at 95°C for 3 min followed by 40 cycles at 95°C for 20 sec, 60°C for 30 sec, and 72°C for 30 sec, in a 25µl reaction volume containing 200nmol/l fluorescect probe and 12.5µl iQ^{TM} supermix (Bio-Rad, Hercules, CA) in triplicate on the iCycler iQTM Real-Time PCR Detection system (Bio-Rad, hercules, CA).
